# Beyond knowledge acquisition: the role of problem-based learning in supporting competency and skills development in pharmacy education — a systematic review

**DOI:** 10.1186/s12909-026-10181-2

**Published:** 2026-08-15

**Authors:** Stephanie Atweh, Yasmeen Eltoum, Tarik Al-Diery

**Affiliations:** Clinical Pharmacy and Practice, College of Pharmacy, QU Health, Qatar University, Doha, Qatar

**Keywords:** Problem-based learning, Self-regulated learning, Metacognition, Pharmacy education, Competency-based education

## Abstract

**Objective:**

To synthesize evidence on the role of PBL in pre-registration pharmacy education in fostering metacognitive and interpersonal competency development, with particular emphasis on self-regulation, learner autonomy, and professional collaboration.

**Methods:**

A systematic search of MEDLINE, Embase, ERIC, Scopus, and Cumulative Index to Nursing and Allied Health Literature (CINAHL) identified peer-reviewed primary studies exploring PBL-related competency and skill development among undergraduate pharmacy students. Quantitative, qualitative, and mixed methods studies were included. Two reviewers independently screened studies and extracted data. Findings were synthesized narratively, and methodological quality was appraised using the Mixed Methods Appraisal Tool 2018.

**Results:**

Twenty-six studies met the inclusion criteria (12 mixed methods, 11 quantitative descriptive, 3 qualitative). Three domains emerged from the narrative synthesis: (1) Self-regulated clinical reasoning, decision-making, and metacognitive development (*n* = 16); (2) Communication and teamwork skills (*n* = 12); and (3) Engagement, motivation, and learning management skills, including organization and accountability (*n* = 9). Across studies, PBL was associated with enhanced reflective capacity, greater ownership of learning, improved confidence in complex decision-making, and strengthened collaborative communication. However, experiences were not uniformly positive; some studies reported learner anxiety, resistance, or reduced engagement when cultural alignment was limited. Most studies were rated medium to high quality.

**Conclusion:**

Beyond cognitive gains, PBL appears to cultivate self-regulation, learner autonomy, and collaborative competencies essential for preparing pharmacists capable of navigating the evolving scope of contemporary practice. Further longitudinal, theory-informed research is needed to clarify the contextual conditions under which PBL most effectively supports sustained learner development.

**Supplementary Information:**

The online version contains supplementary material available at https://doi.org/10.1186/s12909-026-10181-2.

## Introduction

Problem-based learning (PBL) has emerged as a prominent pedagogical approach in health professions education due to its capacity to connect theoretical knowledge with authentic, person-centered clinical problems [1]. As a learner-centered teaching method, PBL uses complex patient case scenarios involving drug-related problems as the foundation for self-directed learning, requiring students to engage in small-group discussions that foster critical thinking, effective communication, and teamwork, ultimately enabling them to develop evidence-based assessments and personalized pharmaceutical care plans [2–4]. In light of these intended benefits, the debate has shifted from whether PBL is effective in supporting trainee development to *when* it is most beneficial for optimizing learning experiences [5].

In contrast to lecture-based learning, PBL facilitates collaborative small-group learning aimed at strengthening cognitive capacity, conceptual reasoning, and critical-thinking skills, while promoting long-term knowledge retention beyond traditional teaching techniques [6–8]. Similarly, when compared with case-based learning, which typically uses structured cases to reinforce predefined learning objectives under greater faculty guidance, PBL places greater emphasis on learner autonomy and problem formulation, potentially fostering stronger self-directed learning and cognitive flexibility [9]. Other pedagogies, such as team-based learning and simulation learning, also support competency development but often target specific skills (collaborative and applied decision-making) or contexts rather than the integrated reasoning processes central to pharmacy practice [10–13].

Over recent decades, the pharmacy profession has transitioned from a product-focused model to a person-centered, team-based practice model [14, 15]. This shift has placed increased expectations on pharmacists to demonstrate leadership and collaboration, system-level thinking and decision-making, intrinsic motivation, and self-reflection skills [16]. However, multiple studies suggest that these competencies are often underdeveloped among pharmacy graduates, limiting the profession’s ability to fully realize its evolving scope of practice [17–19]. Langley and Aheer highlighted that pharmacy employers identified significant weaknesses in graduates’ ability to ‘apply theoretical knowledge into practice and the lack of good communication and interpersonal skills’ [20]. Further research within a Chinese setting noted that pharmacy graduates ‘lack job readiness and clinical soft skills’ [21]. Addressing this gap necessitates purposeful reform, with pharmacy educators serving as key drivers of curricular change [22]. Applying appropriate educational models to guide reform requires clearly articulated objectives and outcomes, with an emphasis on developing transferable competencies and skills rather than knowledge acquisition alone [23, 24].

Across pharmacy curricula, PBL has been associated with improvements in knowledge acquisition and performance-based outcomes, including enhanced academic achievement and examination performance among pharmacy students [25]. While earlier reviews have synthesized the cognitive benefits of PBL, the literature has largely overlooked its influence on broader professional competencies and “soft skills,” such as metacognition, self-directed learning, communication, and teamwork, which are often more difficult to quantify [24, 26]. As pharmacy curricula worldwide increasingly aim to produce practice-ready pharmacists who are autonomous, reflective, and capable of collaborative practice within a rapidly evolving profession [27], a clearer understanding of how PBL supports the development of these transferable skills is needed.

Accordingly, this systematic review synthesizes evidence on the role of PBL in pre-registration pharmacy education in fostering metacognitive and interpersonal competency development. Extending beyond cognitive gains, this review also explores the mechanisms through which PBL may cultivate the skills, attitudes, and learning behaviors required for collaborative practice and lifelong learning. By dedicating the focus to pharmacy learners and foregrounding competency development, this review seeks to provide a comprehensive understanding of how PBL supports the preparation of future pharmacists for contemporary practice.

## Methods

### Research question development and eligibility criteria

This systematic review was conducted in compliance with the Preferred Reporting Items for Systematic Reviews and Meta-Analyses (PRISMA) 2020 guidelines [28] and adhered to the Joanna Briggs Institute (JBI) checklist for systematic reviews and research syntheses [29]. The review protocol was prospectively registered on the International Prospective Register of Systematic Reviews (PROSPERO) (Project ID: CRD420251009010). The Population, Intervention, Outcome (PIO) framework was used to guide the development of the review question, search strategy, and eligibility criteria. An exploratory research design was used to explore how pharmacy students and educators perceived the role of problem-based learning in supporting the development of competencies and skills in undergraduate pharmacy education, including critical thinking, problem-solving, and key soft skills such as communication, teamwork, and engagement. Guided by this framework, the review addressed the following question: what are the perceptions of pharmacy students and educators on how problem-based learning supports competency and skills development among pharmacy students?

For the purpose of this review, PBL was defined as a learner-centered approach in which learning is initiated by a complex clinical problem addressed through small-group discussion, self-directed learning, and tutor facilitation. Studies were included only when the intervention demonstrated core PBL features: student-led problem analysis, identification of learning needs, independent information seeking, and reconvening to apply new knowledge to problem resolution. Case-based and team-based learning interventions were excluded when learning was primarily structured around predefined content or faculty-directed application of knowledge rather than learner-generated inquiry arising from an ill-structured clinical problem.

Eligible studies were those that explicitly reported outcomes related to skills and competency development among undergraduate pharmacy students. Studies were required to assess PBL-related skills and competencies as primary or secondary outcomes, rather than focusing exclusively on knowledge acquisition, retention, or exam performance. Studies involving multiple healthcare disciplines were excluded if pharmacy-specific findings were not reported separately.

The review included published, peer-reviewed full-text studies employing quantitative, qualitative, or mixed methods designs that presented data evaluating the outcomes of PBL in undergraduate pharmacy students. Conference abstracts, review articles, editorials, and commentary articles were excluded to ensure the inclusion of robust and comprehensive empirical evidence. Non-English studies were excluded if an accurate translation was not available. To comprehensively capture all relevant studies evaluating PBL-related competency and skills development in pre-registration pharmacy education, no publication date limits were applied to the inclusion criteria.

### Search strategy

The search strategy was informed by an initial pilot literature review. Articles aligning with the objectives of the review were examined to identify relevant keywords and subject headings, which were then systematically documented and used to develop a comprehensive and sensitive search strategy. In consultation with a university-affiliated academic librarian, subject headings and keywords were refined and initially tested in Ovid MEDLINE. After several iterative refinements, the Ovid MEDLINE search strategy was finalized and then adapted appropriately for each additional database to ensure thorough retrieval of relevant literature.

The electronic search was conducted in English across five databases (MEDLINE, Embase, ERIC, Scopus, and Cumulative Index to Nursing and Allied Health Literature (CINAHL)) in March 2025 and repeated in January 2026 to search for any new studies that had been published since the initial search date. In addition to the electronic database searches, the reference lists of included studies were examined to identify any further potentially eligible articles. No publication date limits were imposed. Given the focus on peer-reviewed full-text articles, a search of grey literature sources such as ProQuest Dissertations and Theses Global was not undertaken. The complete search strategy for all databases is provided in Appendix A.

### Study screening

All studies identified through the database searches were initially imported into EndNote 21 (Clarivate, Philadelphia, PA, USA) for reference management. Duplicate records were not removed at this stage, as all citations were subsequently imported into Covidence (Melbourne, Victoria, Australia), a web-based systematic review management platform, which automatically identified and removed duplicates prior to screening.

Study selection was conducted using a two-stage screening process in Covidence by two independent reviewers (SA and YE). In the first stage, titles and abstracts were screened to assess eligibility against the predefined inclusion criteria. Where abstracts were unavailable or titles and abstracts provided insufficient information, the full text was retrieved for further assessment. In the second stage, full-text articles were reviewed to determine eligibility, specifically assessing whether the studies examined the role of problem-based learning in supporting competency and skills development in undergraduate pharmacy education. Reasons for exclusion were documented at the full-text screening stage. Any disagreements between the two reviewers at either stage of screening were resolved through discussion with a third reviewer (TD).

### Data extraction, analysis, and synthesis

Data extraction was carried out using a customized extraction table developed in Microsoft Word 365 (Microsoft Corp., Redmond, WA, USA). Initial extraction was performed by two reviewers (SA and YE), with the accuracy and completeness of the extracted data independently verified by a third reviewer (TD). The extraction table was designed to systematically capture essential study characteristics, including author and year of publication, country of origin, study aim, study context, type of study, study design, description of participants, and main reported outcomes.

Following data extraction, all members of the research team reviewed the extracted data and collaboratively undertook a narrative synthesis of the included studies. Findings from qualitative, quantitative descriptive, and mixed-methods studies were integrated through a narrative thematic synthesis approach. Key concepts, descriptive terms, and interpretive labels were inductively assigned to summarize the principal findings of each study. Through an iterative process of discussion and comparison, studies demonstrating similar findings were clustered and subsequently organized into broader thematic categories that reflected patterns related to PBL outcomes. Consensus among the research team was reached on the final thematic structure to promote consistency, transparency, and rigor in interpretation.

This analytical approach supported the integration of evidence derived from qualitative, quantitative descriptive, and mixed methods studies, allowing for the identification of common themes across diverse methodological designs and educational contexts while maintaining the integrity and depth of individual study findings.

### Quality assessment

The methodological quality of the included studies was evaluated using the 2018 version of the Mixed Methods Appraisal Tool (MMAT) [30]. This tool was selected for its ability to appraise a broad range of study designs, including qualitative studies, randomized controlled trials, non-randomized studies, quantitative descriptive studies, and mixed methods research. Given the inclusion of studies with diverse methodological approaches and the absence of design restrictions, MMAT offered a comprehensive and appropriate framework for quality assessment.

Quality appraisal was independently undertaken by two reviewers (SA and TD) using the Covidence© platform, with assessment criteria applied according to the specific design of each study. Each study was evaluated against the relevant MMAT methodological criteria, with judgments recorded as “yes,” “no,” or “can’t tell.” For studies employing mixed methods designs, reviewers assessed the qualitative and quantitative descriptive components in addition to the integrated mixed methods criteria. Any discrepancies in quality ratings were addressed through discussion, and consensus was reached between reviewers to ensure consistency and transparency in the appraisal process.

## Results

### Study selection

**Fig. 1:**
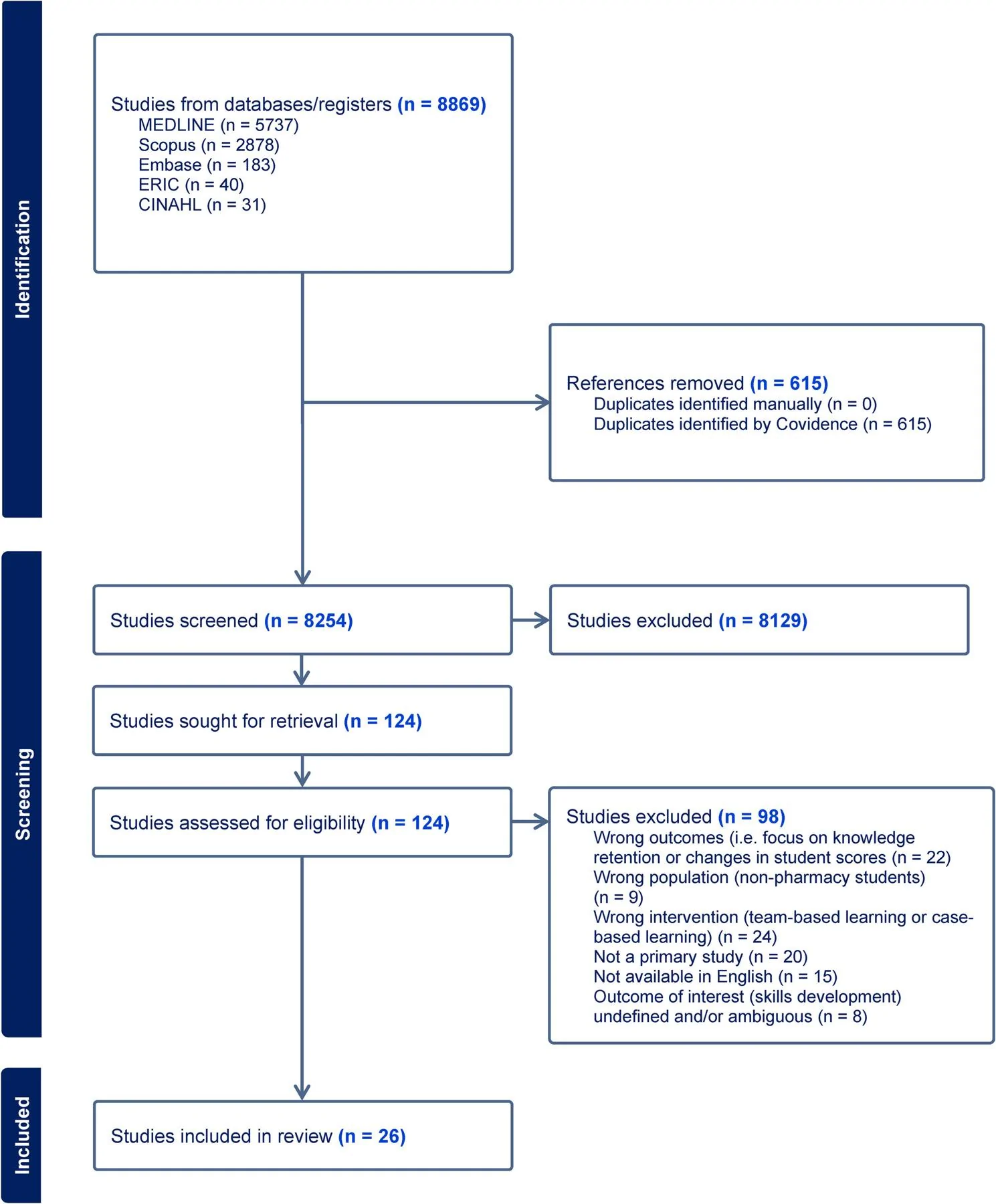
PRISMA flow diagram of the study selection process for studies exploring competency and skills development through PBL in pharmacy education

The search and selection process is presented in the PRISMA flow diagram (Fig. 1). The initial search yielded 8,869 records, of which 615 duplicates were removed. Following title and abstract screening of the remaining 8,254 records, 124 full-text articles were assessed for eligibility. Of these, 26 primary studies met the inclusion criteria, specifically exploring the role of PBL in supporting competency and skills development among pre-registration pharmacy students.

A total of 98 full-text articles were excluded for the following reasons: outcomes not aligned with the review focus (e.g. studies examining knowledge retention or changes in examination performance only), inclusion of non-pharmacy student populations, inappropriate study designs (e.g. reviews, commentaries, and conference abstracts), incorrect educational interventions (e.g. team-based or case-based learning rather than PBL), non-English language, and studies in which competency and skills development outcomes were undefined or insufficiently described.

### Study characteristics

Of the 26 articles that met the inclusion criteria, 12 were mixed methods studies, 11 were quantitative descriptive studies, and three were qualitative studies. Most studies were conducted in the United States (*n* = 8), followed by Canada (*n* = 3) and Malaysia (*n* = 3). Two studies were from Australia, while single studies were conducted in China, Nigeria, Indonesia, Russia, Iran, the United Kingdom, Qatar, and the United Arab Emirates. Across the included studies, PBL was predominantly implemented in classroom-based, small-group setting. A comprehensive summary of the extracted data is provided in Appendix B and summarized in the tables below (Tables 1, 2, and 3).

**Table 1:** Self-regulated clinical reasoning, decision-making, and metacognitive development

Author, Year	Country	Study design	Description of participants	Main outcomes
Abate, 2000 [31]	United States	Mixed methods. Quasi-experimental design.	86 pharmacy students and 10 facilitators.	PBL effectively fostered the development of critical thinking capabilities in students while enhancing collaborative problem-solving.
Azu, 2011 [32]	Nigeria	Quantitative descriptive, cross-sectional comparative survey study.	133 s year students.	82.7% of students agreed or strongly agreed that PBL gave them “ownership” of the problem, allowing them to take responsibility for defining and addressing their own learning needs.
Chen, 2024 [26]	China	Quantitative descriptive parallel-group PBL vs. lecture-based learning post-intervention comparison.	57 third-year pharmacy students.	PBL students demonstrated significantly higher problem-solving, self-directed learning, and critical thinking scores than those in lecture-based learning (problem-solving: 8.43 vs. 7.02, *p* = 0.002; self-directed learning: 7.39 vs. 6.41, *p* = 0.004; critical thinking: 7.96 vs. 6.84, *p* = 0.02).
Duncan-Hewitt, 1996 [33]	United States	Mixed methods. Quasi-experimental pre-post design using student questionnaires and reflective learning journals to evaluate the impact of a structured, process-focused PBL intervention.	224 s- and third-year pharmacy students.	The structured, process-focused PBL approach enhanced metacognition and self-directed learning through the use of reflective journals and systematic self-evaluation.
Ellis, 2008 [34]	Australia	Mixed methods. Qualitative phenomenographic design, followed by quantitative analyses using cross-tabulations.	166 fourth year pharmacy students.	PBL supported the development of clinical reasoning and diagnostic problem-solving skills, whilst facilitating growth in professional judgement and decision-making capabilities.
Ge, 2010 [35]	United States	Mixed methods. Experimental study using a randomized controlled, pre-post design.	96 third-year pharmacy students (49 in the treatment group and 47 in the control group).	Exposure to expert modelling promoted self-reflection which helped students internalize standards of practice and improve self-directed professional development. The support system helped students build metacognitive skills fundamental to professionalism.
Hartayu, 2018 [36]	Indonesia	Mixed methods. Qualitative components included focus group discussions with faculty and preceptors, analysis of students’ reflective journals and supervisors’ observations, alongside performance assessment data.	Unspecified number of participants.	The initiating trigger cases were perceived as realistic and aligned with community pharmacy practice, and were associated with improvements in students’ problem-solving ability, critical thinking, and confidence in clinical decision-making.
Hogan, 2006 [37]	United States	Quantitative descriptive cross-sectional survey.	109 graduating PharmD students. Class of 2004: 69 students. Class of 2005: 40 students.	80% of students reported above-average preparedness in self-directed learning and information literacy following exposure to PBL.
Jones, 2005 [38]	Australia	Qualitative evaluation of PBL using student surveys and reflective self-assessment.	Unspecified	PBL improved students’ ability to be self-directed and manage their own learning (67.6%), whilst also improving their problem-solving skills (61.7%).
Juba, 2014 [39]	United States	Mixed methods. Pre–post quasi-experimental design, with free-response pre- and post-tests, case performance scores, and thematic analysis of student self-reflections.	A total of 25 s- and third-year pharmacy students.	Performance on three individual case studies averaged > 80%, with few recurrent errors, demonstrating students’ ability to develop their clinical reasoning and application skills in complex cases.
Lipari, 2022 [40]	United States	Quantitative descriptive, pre–post observational design.	88 first-year PharmD students.	Students’ self-assessed PBL skills (10 items) improved significantly from the first to the second assessment *P* < 0.001.
Lobuteva, 2019 [41]	Russia	Mixed methods design combing anonymous questionnaire surveys with focus group discussion.	423 students (236 first-year and 187 final-year students).	PBL enhanced the development of critical thinking skills in students, allowing them to apply theoretical knowledge to real-life situations, whilst increasing their professional readiness for life-long and self-directed learning.
Nicholl, 2012 [42]	Canada	Mixed methods. Cross-sectional evaluation, with outcomes assessed based on students’ performance and course evaluation surveys.	239 fourth-year pharmacy students.	The majority of respondents noted that PBL strengthened clinical reasoning and problem solving, whilst promoting them to be self-directed and autonomous in their learning.
Raisch, 1995 [43]	United States	Quantitative descriptive PBL versus traditional learning.	45 fourth-year pharmacy students. 14 facilitators.	PBL improved students’ problem-solving skills, application of didactic knowledge, teamwork, and self-directed learning.
Ravari, 2014 [44]	Iran	Quantitative descriptive quasi-experimental self-control with a pre-post educational intervention design.	73 pharmacy students.	Around two-thirds of students found the modified PBL to improve self-directed learning, comprehension and dynamic problem-solving skills. However, more than half also found it anxiety inducing.
Wright, 2014 [45]	United Kingdom	Mixed methods. Cross-sectional questionnaire with Likert scale and open-ended questions.	201 pharmacy students across four professional year levels.	PBL improved students’ ability to be self-directed and manage their own learning (67.6%).

**Table 2:** Communication, teamwork, and professional collaboration

Author, Year	Country	Study design	Description of participants	Main outcomes
Abate, 2000 [31]	United States	Mixed methods. Quasi-experimental design.	86 pharmacy students and 10 facilitators.	Students reported improved oral communication skills, such as articulating reasoning, asking clarifying questions, and engaging in respectful group dialogue. Facilitator-led sessions provided more opportunities for verbal interaction, enhancing interpersonal communication.
Azu, 2011 [32]	Nigeria	Quantitative descriptive, cross-sectional comparative survey study.	133 s year students.	91.0% agreed that group-based learning enhanced teamwork, collaboration, and discussion skills.
Chen, 2024 [26]	China	Quantitative descriptive parallel-group PBL vs. lecture-based learning post-intervention comparison.	57 third-year pharmacy students.	PBL significantly improved communication skills (8.86 vs. 7.68; *p* = 0.01), with students reporting enhanced collaborative working during small-group discussions.
Choo, 2022 [46]	Malaysia	Mixed methods. Descriptive quantitative analysis of pre- and post-surveys, and qualitative thematic analysis.	169 s-year pharmacy undergraduate students.	Improved cognitive, communication, and teamwork competencies, demonstrated by high levels of cognitive presence and collaborative problem solving in virtual PBL (expert and base group engagement mean scores 4.34–4.88/5).
Curran, 2005 [47]	Canada	Mixed methods. Pre-post-test with open-ended questions.	133 health sciences students: 45 nursing (3rd year), 62 medicine (2nd year), and 26 pharmacy (final year) students.	PBL with standardized patients enhanced information sharing, interprofessional care planning, communication, and respect for professional roles, supporting development of key interprofessional competencies for HIV/AIDS management.
Ferreri, 2013 [48]	United States	Quantitative descriptive quasi-experimental pre–post design, comparing two consecutive cohorts.	150 s-year PharmD students	Students demonstrated enhanced teamwork, communication, and collaborative skills following exposure to PBL.
Jones, 2005 [38]	Australia	Qualitative evaluation of PBL using student surveys and reflective self-assessment.	Unspecified	Most students reported that PBL supported their oral communication (89.1%) and team-working (83.1%) skills.
McKenzie, 2005 [49]	United Kingdom	Qualitative exploratory design using semi-structured interviews with students and focus groups with faculty.	7 s-year and 15 third-year MPharm students. 6 faculty members.	PBL enhanced lifelong learning attitudes and group collaboration but required stronger structure and clearer expectations.
Nasr, 2017 [50]	Qatar	Qualitative exploration and reflective analysis conducted using a SWOC (Strengths, Weaknesses, Opportunities, and Challenges) framework.	Four pharmacy faculty and 75 female BSc (Pharm) students.	PBL enhanced collaboration and teamwork skills amongst students, fostering increased confidence in working as part of larger teams.
Nicholl, 2012 [42]	Canada	Mixed methods. Cross-sectional evaluation, with outcomes assessed based on students’ performance and course evaluation surveys.	239 fourth-year pharmacy students.	Students reported enhanced communication and teamwork skills following exposure to PBL, with better engagement in small-group discussions, peer feedback, and collaborative decision-making.
Poh-Yen, 2017 [51]	Malaysia	Quantitative descriptive experimental one-group design (*n* = 5) with multiple performance assessments across a 14-week research project.	5 s year students and 1 group facilitator.	Students demonstrated significant development in research, teamwork, leadership skills, enhanced engagement, accountability, and reflection.
Winslade, 1994 [52]	Canada	Mixed methods. Pre-post implementation survey and faculty feedback.	160 final-year pharmacy students. 12 trained facilitators.	Faculty and students reported high satisfaction with the PBL format, noting enhanced teamwork and collaboration.

**Table 3:** Engagement, motivation, and learning management skills

Author, Year	Country	Study design	Description of participants	Main outcomes
Chen, 2024 [26]	China	Quantitative descriptive parallel-group PBL vs. lecture-based learning post-intervention comparison.	57 third-year pharmacy students.	PBL students reported significantly higher satisfaction and interest in the course than lecture-based learners (*p* < 0.001) and rated PBL as more engaging and relevant to future practice (mean Likert scores ≈ 3.8–4.0/5).
Ferreri, 2013 [48]	United States	Quantitative descriptive quasi-experimental pre-post design, comparing two consecutive cohorts.	150 s-year PharmD students	Students and faculty perceived higher motivation and accountability in the redesigned PBL course.
Giuliano, 2020 [53]	United States	Mixed methods. Comparative study between two cohorts: traditional PBL (T-PBL, written format) and verbal defense PBL (V-PBL).	82 third-year students in the traditional PBL group and 47 students in the verbal defense PBL group.	Students developed better organizational skills, used more structured approaches to synthesize and explain information, aligning with cognitive organization and time management.
Hamoudi, 2010 [54]	United Arab Emirates	Quantitative descriptive pre-post intervention study.	95 s and third-year Bachelor of Pharmacy students.	Students highlighted that PBL increased their motivation, confidence, and engagement but noted challenges with time management, group participation balance, and facilitator variability.
Hussain, 2019 [55]	Malaysia	Quantitative descriptive quasi-experimental, comparative design with pre- and post-intervention.	264 undergraduate pharmacy students.	A majority of students reported positive emotions (relaxed, motivated, stimulated) after PBL sessions, reflecting increased confidence and engagement.
McKenzie, 2005 [49]	United Kingdom	Qualitative exploratory design using semi-structured interviews with students and focus groups with faculty.	7 s year and 15 third-year MPharm students. 6 faculty members.	PBL enhanced lifelong learning attitudes and group collaboration but required stronger structure and clearer expectations.
Nasr, 2017 [50]	Qatar	Qualitative exploration and reflective analysis conducted using a SWOC (Strengths, Weaknesses, Opportunities, and Challenges) framework.	Four pharmacy faculty and 75 female BSc (Pharm) students.	While PBL enhanced student learning engagement, resistance to active learning among students, and cultural norms (e.g., high power distance and preference for didactic instruction) limited acceptance and effective adoption of PBL.
Poh-Yen, 2017 [51]	Malaysia	Quantitative descriptive experimental one-group design (*n* = 5) with multiple performance assessments across a 14-week research project.	5 s year students and 1 group facilitator.	Students expressed increased independence, motivation and confidence for future research projects.
Ravari, 2014 [44]	Iran	Quantitative descriptive quasi-experimental self-control with a pre-post educational intervention design.	73 pharmacy students.	Students reported that PBL improved their motivation and confidence; however, around half the students found it anxiety inducing.

The narrative synthesis identified three overarching domains through which PBL was reported to support competency and skills development in pre-registration pharmacy students: (1) self-regulated clinical reasoning, decision-making, and metacognitive development (*n* = 16); (2) communication, teamwork, and interprofessional development (*n* = 12); and (3) engagement, motivation, and learning management skills (*n* = 9). Ten studies addressed outcomes spanning more than one domain and were therefore mapped to multiple themes. A quality assessment using MMAT indicated that most studies were of medium to high quality. A detailed breakdown of the quality assessment is presented in Table 4.

**Table 4:** Quality assessment for included articles using the Mixed Methods Assessment tool (corresponding key at the bottom of the table)

Author, Year	*Screening*		*Qualitative studies*					*Quantitative descriptive studies*					*Mixed method studies*				
	S1	S2	1.1	1.2	1.3	1.4	1.5	4.1	4.2	4.3	4.4	4.5	5.1	5.2	5.3	5.4	5.5
Articles reporting qualitative data																	
Jones, 2005 [38]	Y	Y	Y	CT	CT	Y	N										
McKenzie 2017 [49]	Y	Y	Y	Y	Y	Y	Y										
Nasr, 2017 [50]	Y	Y	Y	Y	Y	N	Y										
Articles reporting quantitative descriptive data																	
Azu, 2011 [32]	Y	Y						Y	Y	Y	Y	Y					
Chen,2024 [26]	Y	Y						Y	Y	Y	CT	Y					
Ferreri, 2013 [48]	Y	Y						Y	Y	Y	Y	Y					
Hamoudi, 2010 [54]	Y	Y						Y	CT	Y	CT	Y					
Hogan, 2006 [37]	Y	Y						Y	CT	Y	CT	Y					
Hussain, 2019 [55]	Y	Y						Y	Y	Y	CT	Y					
Lipari, 2022 [40]	Y	Y						Y	Y	Y	Y	Y					
Poh-Yen, 2017 [51]	Y	Y						N	N	Y	Y	CT					
Raisch, 1995 [43]	Y	Y						Y	Y	Y	Y	Y					
Ravari, 2014 [44]	Y	Y						Y	Y	Y	Y	Y					
Winslade, 1994 [52]	Y	Y						Y	Y	Y	Y	Y					
Articles reporting mixed methods data																	
Abate, 2000 [31]	Y	Y	Y	Y	Y	CT	Y	Y	Y	CT	Y	Y	Y	Y	Y	CT	Y
Choo, 2022 [46]	Y	Y	Y	Y	Y	CT	Y	Y	Y	CT	Y	Y	Y	Y	Y	CT	Y
Curran, 2005 [47]	Y	Y	Y	Y	Y	Y	Y	Y	Y	N	Y	Y	Y	Y	Y	Y	Y
Duncan-Hewitt, 1996 [33]	Y	Y	Y	Y	Y	CT	Y	Y	Y	CT	Y	Y	Y	Y	Y	CT	Y
Ellis, 2008 [34]	Y	Y	Y	Y	Y	CT	Y	Y	Y	CT	Y	Y	Y	Y	Y	CT	Y
Ge, 2010 [35]	Y	Y	Y	CT	Y	CT	Y	Y	Y	Y	Y	Y	Y	Y	Y	Y	Y
Giuliano, 2021 [53]	Y	Y	Y	Y	Y	Y	Y	Y	Y	Y	Y	Y	Y	Y	Y	Y	Y
Hartayu, 2018 [36]	Y	Y	Y	Y	Y	Y	Y	Y	Y	Y	CT	Y	Y	Y	Y	Y	Y
Juba, 2014 [39]	Y	Y	CT	CT	Y	N	CT	Y	Y	CT	CT	Y	Y	CT	CT	N	CT
Lobuteva, 2019 [41]	Y	Y	Y	CT	CT	N	CT	Y	Y	CT	Y	Y	N	N	N	N	CT
Nicholl, 2012 [42]	Y	Y	Y	CT	CT	N	CT	Y	Y	CT	Y	Y	N	N	N	N	CT
Wright, 2014 [45]	Y	Y	Y	CT	CT	Y	CT	Y	Y	CT	CT	Y	N	Y	Y	Y	Y

*Y Yes, *N* No, *CT* Cannot tell

Screening questions (for all studies)

S1: Are there clear research questions?

S2: Do the collected data allow to address the research question?

Qualitative studies

Is the qualitative approach appropriate to answer the research question?

Are the qualitative data collection methods adequate to address the research question?

Are the findings adequately derived from the data?

Is the interpretation of the results sufficiently substantiated by data?

Is there coherence between qualitative data sources, collection, analysis and interpretation?

Quantitative descriptive studies

4.1. Is the sampling strategy relevant to address the research question?

4.2. Is the sample representative of the target population?

4.3. Are the measurements appropriate?

4.4. Is the risk of nonresponse bias low?

4.5. Is the statistical analysis appropriate to answer the research question?

Mixed methods studies

5.1. Is there an adequate rationale for using a mixed methods design to address the research question?

5.2. Are the different components of the study effectively integrated to answer the research question?

5.3. Are the outputs of the integration of qualitative and quantitative components adequately interpreted?

5.4. Are divergences and inconsistencies between qualitative and quantitative results adequately addressed?

5.5. Do the different components of the study adhere to the quality criteria of each tradition of the methods involved?

### Self-regulated clinical reasoning, decision-making, and metacognitive development

Critical thinking, decision-making, problem-solving, and self-directed learning skills were reported in 16 studies. Of these, nine were mixed methods studies, six were quantitative descriptive studies, and one was a qualitative study. A summary of the study characteristics and key findings related to self-regulated thinking and metacognitive development are reported in Table 1.

Across the included studies, exposure to PBL was consistently associated with enhanced clinical reasoning, critical thinking, and problem-solving, particularly in the application of didactic knowledge to authentic and complex practice scenarios [26, 31, 34, 36, 38, 41–44]. Students described greater confidence in analyzing problems, generating hypotheses, and justifying decisions, with several studies highlighting improvements in higher-order cognitive processing and clinical judgement [34, 39–42]. Chen and colleagues demonstrated that students in a PBL environment achieved significantly higher problem-solving and critical thinking scores than those taught using lecture-based methods, providing quantitative support for these perceived gains [26].

Beyond cognitive performance, the development of self-regulated learning and metacognitive capability emerged as a central outcome of PBL [26, 32, 33, 35, 37, 38, 41–43, 45]. The learner-centered structure of PBL, characterized by responsibility for defining learning objectives, seeking information, and evaluating one’s own understanding, was reported to promote autonomy, ownership, and reflective practice [33, 35, 42]. Across multiple studies, students described increased awareness of their strengths and limitations, greater capacity to monitor and adjust their learning strategies, and improved confidence in directing their own learning [32, 35, 38, 41, 45]. This metacognitive engagement appeared to underpin the development of self-directedness, with learners becoming more comfortable assuming active responsibility for knowledge construction and decision-making [32, 33, 35, 41, 45].

While the overall pattern indicated positive cognitive and metacognitive development, not all experiences were uniformly favourable. Ravari and colleagues reported that more than half of their cohort experienced heightened anxiety in response to the demands of PBL, suggesting that the cognitive load and responsibility inherent in self-directed, problem-centered learning may be challenging for some students and highlighting the importance of appropriate scaffolding and supervisory support [44].

### Communication, teamwork, and professional collaboration

Twelve studies reported outcomes related to communication, teamwork, and professional collaboration following exposure to PBL, including five mixed methods studies, four quantitative descriptive studies, and three qualitative studies. A summary of the study characteristics and key findings related to communication, teamwork, and interprofessional development are summarized in Table 2.

Collectively, PBL was consistently associated with improvements in students’ communication and discussion skills, particularly in their ability to articulate clinical reasoning, justify decisions, and engage in constructive dialogue [26, 31, 32, 38, 42, 46, 48]. Small-group, case-based interactions provided repeated opportunities for verbal exchange, enabling students to become more confident in expressing viewpoints, asking focused clarifying questions, and engaging respectfully with differing perspectives [31, 32, 42]. Nicholl and colleagues further articulated that PBL facilitated the development of peer feedback skills, highlighting its role in fostering reflective and collaborative discourse [42].

In addition to communication, PBL was reported to cultivate teamwork and collaborative capability [26, 32, 42, 46–52]. Students described enhanced ability for information sharing, joint problem-solving, and consensus-building within diverse groups, alongside increased confidence in participating in and contributing to team-based discussions [26, 49–52]. These experiences supported the development of interpersonal skills essential for collaborative practice, including listening, negotiation, and mutual respect [31, 47, 50]. Extending beyond intraprofessional learning, Curran and colleagues highlighted that interprofessional PBL with standardized patients promoted understanding of professional roles and responsibilities, strengthened interprofessional communication, and fostered respect across disciplines, highlighting the broader value of PBL in preparing students for collaborative healthcare practice [47].

### Engagement, motivation, and learning management skills

Nine studies explored psychosocial and self-regulatory outcomes associated with PBL, including six quantitative descriptive studies, two qualitative studies, and one mixed methods study. A summary of the study characteristics and key findings related to engagement, motivation, and emotional regulation are summarized in Table 3.

Overall, these studies indicated that PBL was associated with increased student engagement and motivation, alongside greater confidence and independence in learner-centered environments [26, 44, 48, 51, 54, 55]. Students described heightened interest in learning, stronger ownership of their educational processes, and increased willingness to actively participate in discussions and problem-solving activities [26, 44, 48, 51, 54]. Chen and colleagues demonstrated significantly higher levels of perceived engagement, interest, and satisfaction among students exposed to PBL compared with those taught using lecture-based methods [26].

Engagement and motivation were often closely linked to improvements in students’ learning management skills, with learners demonstrating greater accountability for their own learning alongside enhanced organizational and time-management abilities [51, 53]. By requiring students to take responsibility for identifying learning needs, planning study activities, and monitoring their progress, PBL fostered a sense of ownership over the learning process [48, 53]. This shift towards greater autonomy and self-management appeared to cultivate the organizational, planning, and accountability skills essential for sustained, self-directed, and lifelong learning [48, 49, 51, 53].

Not all experiences were uniformly positive, however. Two studies highlighted that student engagement and motivation were sensitive to contextual factors, with variability in facilitator expertise and lack of structural clarity within PBL sessions limiting students’ confidence and participation [49, 55]. In addition, studies conducted in Middle Eastern contexts reported that cultural norms, preference for teacher-centered instruction, and resistance to active learning contributed to emotional discomfort and uncertainty, thereby constraining students’ engagement and the full realization of PBL’s intended benefits [44, 50].

## Discussion

This systematic review explored the role of PBL as an effective pedagogy for supporting competency development in pre-registration pharmacy education, particularly in domains that extend beyond knowledge acquisition and academic performance. While earlier literature has emphasized the cognitive benefits of PBL, our findings suggest that PBL also provides a structured learning environment that cultivates metacognitive capacity and interpersonal competence, both of which are essential for developing practice-ready and self-directed pharmacists. Although PBL originated within medical education, these findings reinforce its applicability across health professions training, particularly within competency-based education models [56]. Collectively, while competency-based education defines the intended *outcomes* of training, PBL offers a practical *pedagogical mechanism* through which learners can develop the competencies required to meet those outcomes [57–59]. Through authentic, patient-centered scenarios, PBL mimics the complexity of clinical practice, forcing learners to integrate their theoretical knowledge with critical soft skills [2–5].

Beyond knowledge acquisition and performance-based outcomes, PBL emerged as a powerful platform for fostering critical thinking and self-reflective capacity among pharmacy trainees [31, 33–35, 38]. By positioning learners as active agents responsible for identifying learning needs, exploring clinical questions, and synthesizing solutions under guided facilitation, PBL encourages deeper cognitive engagement beyond surface-level understanding [41, 43]. These findings align closely with principles of cognitive constructivism, whereby learners actively construct meaning by integrating new information into existing cognitive schemas [60]. Rather than passively receiving content, trainees engage in iterative cycles of hypothesis generation, problem refinement, and evidence appraisal within complex, person-centered clinical scenarios. Moreover, PBL provided learners with opportunities to *generate* drug-related problems within complex, person-centered cases and then *refine* these problems into an evidence-based and tailored pharmaceutical care plan. Framed through Zimmerman’s self-regulated learning model, PBL operationalizes the forethought, performance, and self-reflection phases that underpin metacognitive regulation [61]. Across multiple studies in this review, trainees reported greater awareness of their own thinking processes, improved confidence in clinical decision-making, and enhanced ability to evaluate their performance. As lifelong learning is a defining characteristic of collaborative pharmacy practice, the development of critical-thinking and metacognitive capacities can be meaningfully supported through PBL pedagogy [62].

Furthermore, our findings indicate that PBL meaningfully supports the development of transversal “soft” skills, particularly teamwork and interpersonal communication capabilities [31, 38, 46, 51]. Consistent with evidence from nursing and medical education, PBL has been associated with improvements in active listening, structured argumentation, constructive feedback, and overall interaction quality – skills that are essential for effective collaboration in complex clinical environments [63–65]. These findings are particularly relevant in light of the Accreditation Council for Pharmacy Education standards, which mandate the integration of collaborative and interprofessional competencies across the pharmacy curriculum [66]. Development of these competencies begins in the early professional years and is expected to mature throughout experiential training, including Advanced Pharmacy Practice Experiences (APPEs). The studies included in this review spanned learners across multiple stages of the academic continuum, from second-year through final-year students, suggesting that the interpersonal benefits of PBL are not confined to a single phase of training [26, 32, 42, 48, 50]. The inherently interactive structure of PBL – characterized by small-group discussion, peer teaching, and facilitated reflection – creates repeated opportunities for learners to practice communication, presentation, and collaborative decision-making skills. Beyond competency development, sustained peer engagement – both intraprofessionally and interprofessionally – within PBL settings may contribute to the early formation of professional identity, as students begin to situate themselves within a community of practice and internalize the shared values, responsibilities, and norms underpinning collaborative pharmaceutical care [47, 67, 68].

Similarly, early exposure to learner-centered pedagogies such as PBL may enhance students’ motivation and engagement while cultivating the organizational and self-management capacities necessary for autonomous practice [26, 44, 51, 55]. Findings from this review suggest that the structure of PBL encourages learners to assume ownership of goal setting, identify their learning needs, and take accountability for knowledge acquisition; behaviors consistent with self-regulated and self-determined learning [48, 50, 51, 53, 54, 69]. McKenzie and colleagues further suggest that sustained engagement in PBL nurtures lifelong learning attitudes, as learners begin to internalize responsibility for their professional growth [49]. When interpreted through Deci and Ryan’s Self-Determination Theory, these findings can be understood as reflecting the fulfillment of the three basic psychological needs underpinning high-quality motivation: autonomy, competence, and relatedness [69]. PBL supports autonomy by positioning students as active decision-makers in the learning process [32]; it reinforces competence through iterative problem-solving and feedback in complex clinical scenarios [54]; and it fosters relatedness through structured peer collaboration [47]. Collectively, satisfaction of these needs may facilitate a shift from externally regulated motivation (e.g., performance-driven goals) toward more identified or integrated forms of regulation aligned with professional self-development [70, 71]. Across the studies included in this review, learners demonstrated greater initiative in defining learning objectives and improved confidence in managing complex cases – indicators of growing autonomy and perceived competence [72, 73]. In this way, PBL may function not merely as a pedagogical strategy, but as a developmental mechanism that supports the formation of a professional identity grounded in autonomous motivation, collaborative engagement, and sustained commitment to lifelong learning [70].

While PBL embodies a learner-centered teaching approach, the quality of the student experience is highly dependent on facilitator preparedness and the ability to provide effective scaffolding and reinforcement [50, 74]. Transitioning from a traditional “transmission” model of teaching to a facilitative role may be challenging for seasoned faculty accustomed to lecture-based delivery [75]. Furthermore, facilitators in Qatar and Iran have emphasized the importance of culturally adapting PBL strategies to align with students’ prior educational experiences and expectations [44, 50]. In contexts where learning is predominantly theory-driven and passive, PBL has at times generated uncertainty and discomfort among learners, which may hinder engagement [44, 50]. These findings suggest that PBL is not a universally transferable model; rather, its effectiveness depends on contextual adaptation and thoughtful implementation tailored to learner needs.

An additional limitation of the existing literature – and of this review – is the predominance of self-reported measures when evaluating outcomes related to self-directed learning. As articulated by Leary and colleagues, while trainees frequently perceive gains in autonomy and reflective capacity, reliance on self-perception alone constrains conclusions about actual behavioral or performance-based change [70]. Future research should therefore incorporate multimodal evaluation strategies, including facilitator assessments and objective indicators of self-regulated learning behaviors, to more rigorously capture the impact of PBL on learner development.

This review has several notable strengths and limitations. The inclusion of qualitative, quantitative, and mixed methods studies enabled a more comprehensive synthesis of both measurable outcomes and the underlying learning processes associated with PBL. In doing so, this review extends beyond the recent meta-analysis by Chen and colleagues, which quantitatively examined problem-solving, critical thinking, and self-directed learning outcomes but was restricted to randomized controlled trials and predominantly survey-based measures [26]. Although Chen and colleagues’ analysis demonstrated statistically significant cognitive gains [26], it offered limited insight into the mechanisms through which these competencies develop within PBL environments. By contrast, the present review adopts a process-oriented lens, expanding the synthesis beyond cognitive outcomes to examine how PBL cultivates self-regulation, learner autonomy, organizational skills, and accountability – core attributes underpinning self-directed professional practice [69, 70].

Several limitations warrant consideration. First, many included studies relied on self-reported outcomes, which are inherently vulnerable to response and social desirability bias [70]. Second, although studies were drawn from multiple geographic regions, cultural and curricular differences may limit the generalizability of findings [44, 50]. The review was restricted to English-language publications, potentially excluding relevant evidence from other regions and constraining global applicability. Geographic concentration represents another limitation, as nearly half of the included studies (11 of 26) were conducted in North America (United States and Canada). As competency-based education and PBL continue to expand across Asia, Australia, Africa, and other regions, further research is needed to examine the transferability of findings within diverse educational and cultural contexts. Finally, although most studies were rated as medium to high quality, variability in methodological rigor underpins the need for more robust, theory-informed, and longitudinal research evaluating PBL within undergraduate pharmacy curricula worldwide.

## Conclusion

The findings of this review support the integration of problem-based learning not only to enhance academic performance among pharmacy students, but also to cultivate the competencies and dispositions characteristic of self-directed, autonomous, lifelong learners. The evidence reinforces the value of PBL as a pedagogical approach capable of addressing the breadth of outcomes expected within competency-based pharmacy education. Nevertheless, higher-quality, theory-informed, and longitudinal studies are needed to more rigorously evaluate its impact and clarify the extent to which PBL can cultivate knowledge, skills, and attitudes in pharmacy trainees. In particular, future research should examine the optimal intensity and curricular integration of PBL to determine when and how it is most effective across the pharmacy education continuum.

## Supplementary Information

Supplementary Material 1.

Supplementary Material 2.

## Data Availability

All data analyzed during this systematic review are included in this published article and its supplementary information files.

## References

[1] Telang A. Problem-based learning in health professions education: An overview. Arch Med Health Sci. 2014;2(2). 10.4103/2321-4848.144363.

[2] Barrows HS. A taxonomy of problem-based learning methods. Med Educ. 1986;20(6):481–6. 10.1111/j.1365-2923.1986.tb01386.xDigital. 3796328

[3] Dolmans DH, De Grave W, Wolfhagen IH, van der Vleuten CP. Problem-based learning: future challenges for educational practice and research. Med Educ. 2005;39(7):732–41. 10.1111/j.1365-2929.2005.02205.x. 15960794

[4] Schmidt HG, Rotgans JI, Yew EH. The process of problem-based learning: what works and why. Med Educ. 2011;45(8):792–806. 10.1111/j.1365-2923.2011.04035.x. 21752076

[5] Strobel J, van Barneveld A. When is PBL more effective? A meta-synthesis of meta-analyses comparing PBL to conventional classrooms. Interdiscip J Probl Based Learn. 2009;3(1):44–58. 10.7771/1541-5015.1046.

[6] Albanese MA, Mitchell S. Problem-based learning: a review of literature on its outcomes and implementation issues. Acad Med. 1993;68(1):52–81. 10.1097/00001888-199301000-00012. 8447896

[7] Prince KJ, van Mameren H, Hylkema N, Drukker J, Scherpbier AJ, van der Vleuten CP. Does problem-based learning lead to deficiencies in basic science knowledge? An empirical case on anatomy. Med Educ. 2003;37(1):15–21. 10.1046/j.1365-2923.2003.01402.x. 12535111

[8] Vernon DT, Blake RL. Does problem-based learning work? A meta-analysis of evaluative research. Acad Med. 1993;68(7):550–63. 10.1097/00001888-199307000-00015. 8323649

[9] Srinivasan M, Wilkes M, Stevenson F, Nguyen T, Slavin S. Comparing problem-based learning with case-based learning: effects of a major curricular shift at two institutions. Acad Med. 2007;82(1):74–82. 10.1097/01.ACM.0000249963.93776.aa. 17198294

[10] Koster A, Schalekamp T, Meijerman I. Implementation of Competency-Based Pharmacy Education (CBPE). Pharm (Basel). 2017;5(1). 10.3390/pharmacy5010010. PMC5419394 28970422

[11] Nelson M, Allison SD, McCollum M, Luckey SW, Clark DR, Paulsen SM, et al. The Regis Model for pharmacy education: A highly integrated curriculum delivered by Team-Based Learning™ (TBL). Curr Pharm Teach Learn. 2013;5(6):555–63. 10.1016/j.cptl.2013.07.002.

[12] McBane S, Alavandi P, Allen S, Bingham A, Dang Y, Elmes A, et al. Overview of implementation and learning outcomes of simulation in pharmacy education. J Am Coll Clin Pharm. 2023;6(5):528–54. 10.1002/jac5.1784.

[13] Al-Diery T, Hejazi T, Al-Qahtani N, ElHajj M, Rachid O, Jaam M. Evaluating the use of virtual simulation training to support pharmacy students’ competency development in conducting dispensing tasks. Curr Pharm Teach Learn. 2024;16(12):102199. 10.1016/j.cptl.2024.102199. 39241581

[14] Chisholm-Burns MA, Kim Lee J, Spivey CA, Slack M, Herrier RN, Hall-Lipsy E, et al. US pharmacists’ effect as team members on patient care: systematic review and meta-analyses. Med Care. 2010;48(10):923–33. 10.1097/MLR.0b013e3181e57962. 20720510

[15] Al-Diery T, Hussain FN. Person-centred care in pharmacy: time to walk the talk. J Pharm Policy Pract. 2025;18(1):2511152. 10.1080/20523211.2025.2511152. PMC12128123 40458209

[16] Garrett J, Alman M, Gardner S, Born C. Assessing Students’ Metacognitive Skills. Am J Pharm Educ. 2007;71(1). 10.1016/S0002-9459(24)03180-2. PMC1847545 17429514

[17] Frenzel J, Eukel H, Neville M, Castillo S, Donohoe K, Jones M, et al. Essential skills for pharmacy graduates reported by advanced pharmacy practice experience preceptors. Curr Pharm Teach Learn. 2015;7(5):700–4. 10.1016/j.cptl.2015.06.005.

[18] Medina MS, Plaza CM, Stowe CD, Robinson ET, DeLander G, Beck DE, et al. Center for the Advancement of Pharmacy Education 2013 educational outcomes. Am J Pharm Educ. 2013;77(8):162. 10.5688/ajpe778162. PMC3806946 24159203

[19] Al-Diery T, Atweh S, Odeh M. Empowering early career pharmacists: Unleashing non-clinical competencies through pharmacy residency. Curr Pharm Teach Learn. 2024;16(6):411–21. 10.1016/j.cptl.2024.03.015. 38594173

[20] Langley CA, Aheer S. Do pharmacy graduates possess the necessary professional skills? Pharm Educ. 2010;10(2):114–8.

[21] Wang T, Liang J, Chen L, Li S, Zhu M, Cai Y, et al. Enhancing professional adaptability and clinical soft skills of new clinical pharmacists through a pre-training model in China: a randomized controlled trial. BMC Med Educ. 2025;25(1):1722. 10.1186/s12909-025-07951-9. PMC12751867 41466254

[22] Johnson JL, Arif S, Bloom TJ, Isaacs AN, Moseley LE, Janke KK. Preparing Pharmacy Educators as Expedition Guides to Support Professional Identity Formation in Pharmacy Education. Am J Pharm Educ. 2023;87(1):ajpe8944. 10.5688/ajpe8944. PMC10159609 35121571

[23] Cisneros RM, Salisbury-Glennon JD, Anderson-Harper HM. Status of problem-based learning research in pharmacy education: a call for future research. Am J Pharm Educ. 2002;66(1):19–26.

[24] Galvao TF, Silva MT, Neiva CS, Ribeiro LM, Pereira MG. Problem-based learning in pharmaceutical education: a systematic review and meta-analysis. ScientificWorldJournal. 2014;2014. 10.1155/2014/578382 PMC3950357 24701178

[25] Liu L, Du X, Zhang Z, Zhou J. Effect of problem-based learning in pharmacology education: A meta-analysis. Stud Educ Eval. 2019;60:43–58. 10.1016/j.stueduc.2018.11.004.

[26] Chen T, Zhao Y-J, Huang F-Q, Liu Q, Li Y, Alolga RN, et al. The effect of problem-based learning on improving problem-solving, self-directed learning, and critical thinking ability for the pharmacy students: A randomized controlled trial and meta-analysis. PLoS One. 2024;19(12):e0314017. 10.1371/journal.pone.0314017. PMC11611184 39621602

[27] Al-Diery T, Marotti S, Sim YT, Rowett D, Johnson JL. Entrustment in Action: Factors that Contribute to Entrustment Decision-Making Through the Lens of the Supervisor and the Intern. Am J Pharm Educ. 2025;89(9):101478. 10.1016/j.ajpe.2025.101478. 40721195

[28] Page MJ, McKenzie JE, Bossuyt PM, Boutron I, Hoffmann TC, Mulrow CD, et al. The PRISMA 2020 statement: an updated guideline for reporting systematic reviews. BMJ. 2021;372:n71. 10.1136/bmj.n71. PMC8005924 33782057

[29] Aromataris E, Fernandez R, Godfrey CM, Holly C, Khalil H, Tungpunkom P. Summarizing systematic reviews: methodological development, conduct and reporting of an umbrella review approach. JBI Evid Synth. 2015;13(3). 10.1097/XEB.0000000000000055. 26360830

[30] Hong QN, Pluye P, Fàbregues S, Bartlett G, Boardman F, Cargo M et al. Mixed methods appraisal tool (MMAT), version 2018. *Registration of copyright*. 2018;1148552(10):1–7.

[31] Abate MA, Meyer-Stout PJ, Stamatakis MK, Gannett PM, et al. Development and evaluation of computerized problem-based learning cases emphasizing basic sciences concepts. Am J Pharm Educ. 2000;64(1):74.

[32] Azu O, Osinubi A. A survey of problem-based learning and traditional methods of teaching anatomy to 200 level pharmacy students of the University of Lagos. Nigeria Afr J Pharm Pharmacol. 2011;5(2):219–24. 10.5897/AJPP10.382.

[33] Duncan-Hewitt WC. A focus on process improves problem-based learning outcomes in large classes. Am J Pharm Educ. 1996;60(4):408–16.

[34] Ellis RA, Goodyear P, Brillant M, Prosser M. Student experiences of problem-based learning in pharmacy: conceptions of learning, approaches to learning and the integration of face-to-face and on-line activities. Adv Health Sci Educ Theory Pract. 2008;13(5):675–92. 10.1007/s10459-007-9073-3. 17624599

[35] Ge X, Planas LG, Er N. A cognitive support system to scaffold students’ problem-based learning in a web-based learning environment. Interdiscip J Probl Based Learn. 2010;4(1):4. 10.7771/1541-5015.1093.

[36] Hartayu TS, Wijoyo J, Donowati M. Problem-based learning in reflective pedagogy paradigm: Innovative learning in pharmaceutical care. J Young Pharm. 2018;10(2s):S1. 10.5530/jyp.2018.2s.1.

[37] Hogan S, Lundquist LM. The impact of problem-based learning on students’ perceptions of preparedness for advanced pharmacy practice experiences. Am J Pharm Educ. 2006;70(4):82. 10.5688/aj700482. PMC1636981 17136201

[38] Jones KM. The application of problem-based learning to the teaching of microbiology for pharmacy students. Pharm Educ. 2005;5(1).

[39] Juba KM, Ricca BP. Design of a problem-based learning pain and palliative care elective course. Curr Pharm Teach Learn. 2014;6(3):421–8. 10.1016/j.cptl.2014.02.005.

[40] Lipari M, Wilhelm SM, Giuliano CA, Martirosov AL, Salinitri FD. A scaffolded problem-based learning course for first-year pharmacy students. Curr Pharm Teach Learn. 2022;14(3):352–8. 10.1016/j.cptl.2022.01.016. 35307096

[41] Lobuteva A, Lobuteva L, Zakharova O, Krivosheev S, Yermolaeva A. Specifics of problem-based learning in the pharmaceutical education process. J Adv Pharm Edu Res. 2019;9(2):131–6.

[42] Nicholl TA, Lou K. A Model for Small-Group Problem-Based Learning in a Large Class Facilitated by One Instructor. Am J Pharm Educ. 2012;76(6):117. 10.5688/ajpe766117. PMC3425932 22919093

[43] Raisch DW, Holdsworth MT, Mann P, Kabat HF. Incorporating problem-based student-centered learning into pharmacy externship rotations. Am J Pharm Educ. 1995;59:265–265.

[44] Ravari EMH, Nouhi E. Problem-based learning in medicinal chemistry laboratory from pharmacy students’ perspective. Indian J Pharm Educ. 2014;48(2):4–8.

[45] Wright D, Wickham J, Sach T. Problem-based learning: an exploration of student opinions on its educational role in one UK pharmacy undergraduate curriculum. Int J Pharm Pract. 2013;22(3):223–30. 10.1111/ijpp.12060. 23937188

[46] Choo C, Fahrni M, Long C. Analysis of students’ cognitive presence and perception in a custom-designed virtual problem based learning assignment. Int J Emerg Technol Learn. 2022;17(22):132–43.

[47] Curran VR, Mugford JG, Law RMT, Macdonald S. Influence of an Interprofessional HIV/AIDS Education Program on Role Perception, Attitudes and Teamwork Skills of Undergraduate Health Sciences Students. Educ Health. 2005;18(1). 10.1080/13576280500042606 15804644

[48] Ferreri SP, O’Connor SK. Redesign of a Large Lecture Course Into a Small-Group Learning Course. Am J Pharm Educ. 2013;77(1):13. 10.5688/ajpe77113. PMC3578326 23459199

[49] McKenzie B, Brown A. Exploring a Problem-Based Learning Approach in Pharmaceutics. Pharmacy. 2017;5(3):53. 10.3390/pharmacy5030053. PMC5622365 28970465

[50] Nasr ZG, Wilby KJ. Introducing problem-based learning into a Canadian-accredited Middle Eastern educational setting. Curr Pharm Teach Learn. 2017;9(4):719–22. 10.1016/j.cptl.2017.03.027. 29233448

[51] Poh-Yen K. Training of Novice Researchers: A Study on the Outcomes and Benefits of Using the Problem-based Laboratory Approach as an Alternative in the Pharmacy Curriculum. Indian J Pharm Educ. 2017;51(3):355–64.

[52] Winslade N. Large group problem-based learning: a revision from traditional to pharmaceutical care-based therapeutics. Am J Pharm Educ. 1994;58(1):64–72.

[53] Giuliano C, Martirosov AL, Lipari M, Wilhelm S, Salinitri F, Lahiri M, et al. Incorporating verbal defense into problem-based learning. Curr Pharm Teach Learn. 2021;13(2):109–15. 10.1016/j.cptl.2020.05.014. 33454065

[54] Hamoudi NM, Nagavi B, Al-Azzawi AMJ. Problem based learning and its impact on learning behavior of pharmacy students in RAK medical and Health Sciences University. Indian J Pharm Educ. 2010;6:8.

[55] Hussain M, Sahudin S, Abu Samah NH, Anuar NK. Students perception of an industry based approach problem based learning (PBL) and their performance in drug delivery courses. Saudi Pharm J. 2019;27(2):274–82. 10.1016/j.jsps.2018.11.009. PMC6362177 30766440

[56] Sistermans IJ. Integrating competency-based education with a case-based or problem-based learning approach in online health sciences. Asia Pac Educ Rev. 2020;21(4):683–96. 10.1007/s12564-020-09658-6.

[57] International Pharmaceutical Federation (FIP). Competency-based education in pharmacy and pharmaceutical sciences: A FIP handbook to support implementation of competency-based education and training. 2022. Available from: https://www.fip.org/file/5338. Accessed on 22/01/2026.

[58] International Pharmaceutical Federation (FIP). Global competency framework for educators and trainers in pharmacy 2023. Available from: https://www.fip.org/file/5546. Accessed on 22/01/2026.

[59] Al-Haqan A, Waheedi S, Abdullah I, Meilianti S, Shaaban J. Competency development for pharmacy: adopting and adapting the FIP global advanced development framework. Front Med. 2024;11. 10.3389/fmed.2024.1442643. PMC11349709 39206168

[60] Kalina C, Powell K. Cognitive and social constructivism: Developing tools for an effective classroom. Education. 2009;130(2):241–50.

[61] Zimmerman BJ. Becoming a Self-Regulated Learner: An Overview. Theory Pract. 2002;41(2):64–70. 10.1207/s15430421tip4102_2.

[62] Nguyen VPV, Fernandez NA, Benson H. Enhancing lifelong learning skills in pharmacy education: A scoping review of the literature. Curr Pharm Teach Learn. 2026;18(3):102529. 10.1016/j.cptl.2025.102529. 41308402

[63] Allert C, Dellkvist H, Hjelm M, Andersson EK. Nursing students’ experiences of applying problem-based learning to train the core competence teamwork and collaboration: An interview study. Nurs Open. 2022;9(1):569–77. 10.1002/nop2.1098. PMC8685840 34664786

[64] Trullàs JC, Blay C, Sarri E, Pujol R. Effectiveness of problem-based learning methodology in undergraduate medical education: a scoping review. BMC Med Educ. 2022;22(1):104. 10.1186/s12909-022-03154-8. PMC8851721 35177063

[65] Kong L-N, Qin B, Zhou Y-q, Mou S-y, Gao H-M. The effectiveness of problem-based learning on development of nursing students’ critical thinking: A systematic review and meta-analysis. Int J Nurs Stud. 2014;51(3):458–69. 10.1016/j.ijnurstu.2013.06.009. 23850065

[66] Accreditation Council for Pharmacy Education (ACPE). Accreditation standards and key elements for the professional program in pharmacy leading to the doctor of pharmacy degree (Standards 2025). 2025. Available from: www.acpe-accredit.org. Accessed on 20/01/2026.

[67] Tan CP, Van der Molen HT, Schmidt HG. To what extent does problem-based learning contribute to students’ professional identity development? Teach Teach Educ. 2016;54:54–64. 10.1016/j.tate.2015.11.009.

[68] Macdonald D, Isaacs G. Developing a Professional Identity through Problem-Based Learning. Teach Educ. 2001;12(3):315–33. 10.1080/10476210120096579.

[69] Deci EL, Ryan RM. Self-determination theory: A macrotheory of human motivation, development, and health. Can Psychol. 2008;49(3):182–5. 10.1037/a0012801.

[70] Leary H, Walker A, Lefler M, Kuo YC. Self-directed learning in problem‐based learning: a literature review. New Jersey, United States: John Wiley & Sons, Inc.; 2019. pp. 181–98.

[71] Wijnen M, Loyens SMM, Wijnia L, Smeets G, Kroeze MJ, Van der Molen HT. Is problem-based learning associated with students’ motivation? A quantitative and qualitative study. Learn Environ Res. 2018;21(2):173–93. 10.1007/s10984-017-9246-9.

[72] Manuaba IBAP, No Y, Wu C-C. The effectiveness of problem based learning in improving critical thinking, problem-solving and self-directed learning in first-year medical students: A meta-analysis. PLoS One. 2022;17(11):e0277339. 10.1371/journal.pone.0277339. PMC9681085 36413532

[73] Stentoft D. Problem-based projects in medical education: extending PBL practices and broadening learning perspectives. Adv Health Sci Educ Theory Pract. 2019;24(5):959–69. 10.1007/s10459-019-09917-1. 31641941

[74] Lim WK. Problem Based Learning in Medical Education: Handling Objections and Sustainable Implementation. Adv Med Educ Pract. 2023;14:1453–60. 10.2147/amep.S444566. PMC10758192 38164409

[75] Haworth IS, Eriksen SP, Chmait SH, Matsuda LS, McMillan PA, King EA, et al. A Problem Based Learning, Case Study Approach to Pharmaceutics: Faculty and Student Perspectives. Am J Pharm Educ. 1998;62(4):398–405. 10.1016/S0002-9459(24)02030-8.

