# Supplementary material for: Beyond knowledge acquisition: the role of problem-based learning in supporting competency and skills development in pharmacy education — a systematic review
Source: BMC Med Educ. 2026 Aug 15;26:1836. doi: 10.1186/s12909-026-10181-2 (PMC13753360; doi:10.1186/s12909-026-10181-2)
Supplement: Supplementary file 1 [file 12909_2026_10181_MOESM1_ESM.docx]

**Appendix A: Systematic review search strategy across five databases with the corresponding PICO layout**

PICO strategy

**Population**: Pharmacy students, pharmacists, pharmacy educators

**Intervention:** Problem-based learning

**Comparison**: N/A or traditional teach methods

**Outcome:** Problem-based learning, metacognition, critical thinking, teamwork, professionalism, communication skills

| **Concept** | **Key words** | **Subject headings (Ovid)** |
| --- | --- | --- |
| **Population**: | - Pharmacy - Pharmacy students - Pharmacy education | - Pharmacy* - Students, Pharmacy - Students, Pharmacy* - Education, Pharmacy* |
| **Intervention:** Problem-based learning | - PBL - Problem-based learning - Student training - Learner-centred teaching | - Problem-Based Learning* - Problem-Based Learning / methods - Problem-Based Learning* / methods - Problem-Based Learning / methods* - Problem-Based Learning / standards* - Problem-Based Learning* / trends - Education, Pharmacy / methods* - Teaching / methods - Models, Educational - Self-Directed Learning as Topic - Feedback |
| **Outcome:** Evaluation outcomes | - Approaches to learning - Conceptions of learning - Academic performance - Educational effect - Self-assessment - Peer assessment - Metacognition - Critical-thinking - Problem-solving - Self-reflection | - Professional Competence - Clinical Competence - Clinical Competence / standards* - Educational Measurement - Educational Measurement* - Educational Measurement / methods - Educational Measurement / standards* - Problem Solving - Problem Solving* - Thinking* - Decision Making - Interpersonal Relations - Communication - Cooperative Behavior - Health Knowledge, Attitudes, Practice - Personal Satisfaction - Feedback |

**Search Phrases**

Population

- (Pharmacy OR Pharmacy students OR Pharmacy education)

Intervention

- (PBL OR Problem-based learning OR Student training OR Learner-centred teaching)

Outcome

- (Approaches to learning OR Conceptions of learning OR Academic performance OR Educational effect OR Self-assessment OR Peer assessment OR Metacognition OR Critical-thinking OR Problem-solving OR Self-reflection)

**Search Conducted On:** 12^th^ March 2025

**Six Databases Searched:** MEDLINE, Embase, CINAHL, ERIC and SCOPUS

**MEDLINE**

1. ((Pharmacy) OR (Pharmacy students)) OR (Pharmacy education)
2. (((PBL) OR (Problem-based learning)) OR (Student training)) OR (Learner-centred teaching)
3. (((((((((Approaches to learning) OR (Conceptions of learning)) OR (Academic performance)) OR (Educational effect)) OR (Self-assessment)) OR (Peer assessment)) OR (Metacognition)) OR (Critical-thinking)) OR (Problem-solving)) OR (Self-reflection)

Combined To Main Phrase:

4. ((((Pharmacy) OR (Pharmacy students)) OR (Pharmacy education)) AND ((((PBL) OR (Problem-based learning)) OR (Student training)) OR (Learner-centred teaching))) AND ((((((((((Approaches to learning) OR (Conceptions of learning)) OR (Academic performance)) OR (Educational effect)) OR (Self-assessment)) OR (Peer assessment)) OR (Metacognition)) OR (Critical-thinking)) OR (Problem-solving)) OR (Self-reflection))

Results: 5737 Articles

**Embase**

1. 'pharmacy'/exp OR pharmacy OR 'pharmacy students'/exp OR 'pharmacy students' OR 'pharmacy education'/exp OR 'pharmacy education'
2. pbl OR 'problem-based learning' OR 'student training' OR 'learner-centred teaching'
3. 'approaches to learning' OR 'conceptions of learning' OR 'academic performance' OR 'educational effect' OR 'self assessment' OR 'peer assessment' OR metacognition OR 'critical thinking' OR 'problem solving' OR 'self reflection'

Combined To Main Phrase:

4. #1 AND #2 AND #3

Results: 183 Articles

**CINAHL**

1. (Pharmacy OR Pharmacy students OR Pharmacy education)
2. (PBL OR Problem-based learning OR Student training OR Learner-centred teaching)
3. (Approaches to learning OR Conceptions of learning OR Academic performance OR Educational effect OR Self-assessment OR Peer assessment OR Metacognition OR Critical-thinking OR Problem-solving OR Self-reflection)

Combined To Main Phrase:

4. (Pharmacy OR Pharmacy students OR Pharmacy education) AND (PBL OR Problem-based learning OR Student training OR Learner-centred teaching) AND (Approaches to learning OR Conceptions of learning OR Academic performance OR Educational effect OR Self-assessment OR Peer assessment OR Metacognition OR Critical-thinking OR Problem-solving OR Self-reflection)

Results: 31 Articles

**ERIC**

1. (Pharmacy OR Pharmacy students OR Pharmacy education)
2. (PBL OR Problem-based learning OR Student training OR Learner-centred teaching)
3. (Approaches to learning OR Conceptions of learning OR Academic performance OR Educational effect OR Self-assessment OR Peer assessment OR Metacognition OR Critical-thinking OR Problem-solving OR Self-reflection)

Combined To Main Phrase:

4. (Pharmacy OR Pharmacy students OR Pharmacy education) AND (PBL OR Problem-based learning OR Student training OR Learner-centred teaching) AND (Approaches to learning OR Conceptions of learning OR Academic performance OR Educational effect OR Self-assessment OR Peer assessment OR Metacognition OR Critical-thinking OR Problem-solving OR Self-reflection)

Results: 40 Articles

**SCOPUS**

1. pharmacy OR "Pharmacy students" OR "Pharmacy education"
2. pbl OR "Problem-based learning" OR "Student training" OR "Learner-centred teaching"
3. "Approaches to learning" OR "Conceptions of learning" OR "Academic performance" OR "Educational effect" OR self-assessment OR "Peer assessment" OR metacognition OR critical-thinking OR problem-solving OR self-reflection

Combined To Main Phrase:

4. ( pharmacy OR "Pharmacy students" OR "Pharmacy education" ) AND ( pbl OR "Problem-based learning" OR "Student training" OR "Learner-centred teaching" ) AND ( "Approaches to learning" OR "Conceptions of learning" OR "Academic performance" OR "Educational effect" OR self-assessment OR "Peer assessment" OR metacognition OR critical-thinking OR problem-solving OR self-reflection )

Results: 2878 Articles
