# Supplementary material for: Beyond knowledge acquisition: the role of problem-based learning in supporting competency and skills development in pharmacy education — a systematic review
Source: BMC Med Educ. 2026 Aug 15;26:1836. doi: 10.1186/s12909-026-10181-2 (PMC13753360; doi:10.1186/s12909-026-10181-2)
Supplement: Supplementary file 2 [file 12909_2026_10181_MOESM2_ESM.docx]

**Appendix B: Summary of the key findings from the literature for non-clinical skills and competencies gained from problem-based learning (PBL)**

| **First author, Year** | **Country** | **Type of study** | **Study design** | **Description of participants** | **Study aim** | **Context of study** | **Main outcomes** |
| --- | --- | --- | --- | --- | --- | --- | --- |
| **Abate, 2000** | United States | Mixed methods | Quasi-experimental design with control (facilitator-led PBL) and experimental (computerized PBL) groups.  . | 86 pharmacy students and 10 facilitators. | To develop and evaluate computerized PBL cases that integrate basic science concepts (medicinal chemistry and pharmaceutics), and to compare their effectiveness with traditional facilitator-led PBL sessions. | Conducted at West Virginia University School of Pharmacy, where PBL had been used in basic science courses. Surveys covered 3 years of implementation (1997-2000). The study aimed to reduce faculty workload while maintaining the educational benefits of PBL by using computerized cases, some of which included concept maps. | - Both PBL formats effectively supported the development of communication skills, teamwork, critical thinking, and self-directed learning. - Students reported improved oral communication skills, such as articulating reasoning, asking clarifying questions, and engaging in respectful group dialogue. - Written communication was fostered through structured tasks like hypothesis formulation and action planning. - Facilitator-led sessions provided more opportunities for verbal interaction, enhancing interpersonal communication and collaborative problem-solving. |
| **Azu, 2011** | Nigeria | Quantitative descriptive | Quantitative, cross-sectional comparative survey study, in which the same cohort of students evaluated PBL and traditional teaching after exposure to both methods. | 133 second year students. | To compare PBL with traditional teaching methods in anatomy and to evaluate their effects on pharmacy students’ learning behaviours, perceptions, and skill development. | Conducted in the Faculty of Pharmacy at the University of Lagos during the second-semester anatomy course in the 2007–2008 academic year, where PBL was newly introduced alongside traditional lecture-based teaching. Students were divided into fifteen small groups of ten for PBL sessions, facilitated by tutors. | - 82.7% of students agreed or strongly agreed that PBL gave them “ownership” of the problem, allowing them to take responsibility for defining and addressing their own learning needs. - 93.2% of students agreed or strongly agreed that PBL helped them develop the skills needed for problem-solving. - 91.0% agreed that group-based learning enhanced teamwork, collaboration, and discussion skills. - 69.2% believed PBL better prepared them for the pharmacy profession (despite traditional teaching being preferred for exam preparation). |
| **Chen, 2024** | China | Quantitative descriptive | Parallel-group (PBL vs lecture-based learning), post-intervention comparison. | 57 third-year pharmacy students. | To evaluate the effectiveness of PBL on pharmacy students’ problem-solving, self-directed learning, and critical thinking. | Third-year pharmacy students at China Pharmaceutical University enrolled in the “Authentication of Chinese Medicines” course, where a 17-week PBL intervention was compared with lecture-based teaching to develop problem-solving, self-directed learning, and critical thinking. | - PBL students demonstrated significantly higher problem-solving, self-directed learning, and critical thinking scores than those in lecture-based learning (problem-solving: 8.43 vs 7.02, p = 0.002; self-directed learning: 7.39 vs 6.41, p = 0.004; critical thinking: 7.96 vs 6.84, p = 0.02). - PBL significantly improved communication skills (8.86 vs 7.68; p = 0.01), with students reporting enhanced collaborative working during small-group discussions. - PBL students reported significantly higher satisfaction and interest in the course than lecture-based learners (p < 0.001) and rated PBL as more engaging and relevant to future practice (mean Likert scores ≈ 3.8–4.0/5). |
| **Choo, 2022** | Malaysia | Mixed methods | Descriptive quantitative analysis of pre- and post-surveys, and qualitative thematic analysis of student discussions on Jamboard. | 169 second-year pharmacy undergraduate students. | To evaluate pharmacy students' cognitive presence and perception of engagement during a virtual PBL assignment using the Jigsaw model. | Conducted as part of the Pharmaceutical Analysis course at Universiti Teknologi MARA, Malaysia, during the COVID-19 pandemic when all teaching was delivered online. A custom-designed virtual PBL assignment using a Jigsaw cooperative learning model and Jamboard was implemented to support student engagement and cognitive presence within a Community of Inquiry framework. | - Jigsaw PBL promoted collaboration, analytical skills, and a strong sense of community in virtual settings. - Improved cognitive, communication, and teamwork competencies, demonstrated by high levels of cognitive presence and collaborative problem solving in virtual PBL (expert and base group engagement mean scores 4.34–4.88/5). - Peer and facilitator support was critical |
| **Curran, 2005** | Canada | Mixed methods | One-group pre-post implementation and time-series design. Checklist and open-ended response survey questions. | 133 health sciences students: 45 nursing (3rd year), 62 medicine (2nd year), and 26 pharmacy (final year). Each small group (8–10 students) included representatives from all three disciplines. | To evaluate the impact of an interprofessional education program on undergraduate health sciences students’ role perceptions, attitudes toward teamwork, and self-reported teamwork skills using a PBL and standardized patient format. | Conducted at Memorial University of Newfoundland, involving the Faculty of Medicine, School of Pharmacy, and School of Nursing. Students participated in a structured, small-group, PBL-based HIV/AIDS module. | - Pharmacy students’ Weekly Team Inventory (WTI) scores significantly increased over the three sessions (p < 0.001), similar to the overall group trend. They reported greater confidence in communicating and working collaboratively. - PBL with standardized patients enhanced information sharing, interprofessional care planning, communication, and respect for professional roles, supporting development of key interprofessional competencies for HIV/AIDS management. |
| **Duncan-Hewitt 1996** | United States | Mixed methods | Quasi-experimental pre-post design using student questionnaires and reflective learning journals to evaluate the impact of a structured, process-focused PBL intervention. | 224 second- and third-year pharmacy students. | To determine whether introducing a process-focused, structured dialogue approach within a large, tutor-less PBL pharmaceutics course would improve students’ learning outcomes, confidence, enjoyment, and development of problem-solving, critical thinking, self-assessment, and self-directed learning skills, compared with a less structured PBL format. | Conducted at the Texas Tech University’s College of Pharmacy in a pharmaceutics course covering complex and novel drug delivery systems. The course involved second- and third-year pharmacy students working in small groups to solve authentic formulation and drug delivery problems. | - The structured, process-focused PBL approach enhanced metacognition and self-directed learning through the use of reflective journals and systematic self-evaluation. - Students reported strengthened development of critical thinking, communication, and leadership skills through structured group roles and written, instructor-guided dialogue. - Improvements were also observed in time management, organization, and willingness to take intellectual risks, leading to greater confidence and perceived learning gains. |
| **Ellis, 2008** | Australia | Mixed methods | Qualitative phenomenographic design using 15 semi-structured interviews and 166 open-ended questionnaires, followed by quantitative analyses using cross-tabulations. | 166 fourth year pharmacy students. | To investigate pharmacy students’ PBL experiences by examining the relationships between their conceptions of PBL, approaches to learning (face-to-face and online), and academic performance. | Fourth-year undergraduate pharmacy students at the University of Sydney focused on medication review and professional decision-making, delivered using a PBL curriculum. | - PBL supported the development of clinical reasoning and diagnostic problem-solving skills, facilitated growth in professional judgement and decision-making capabilities, and supported self-directed learning in pharmacy students. - PBL was effective in supporting general transferable skills such as teamwork and communication, with students becoming better at cooperating and resolving cases collectively as a unit. - Students who conceptualized PBL as a means for understanding and professional reasoning demonstrated higher competence than those who demonstrated surface-level procedural engagement with PBL. |
| **Ferreri, 2013** | United States | Quantitative descriptive | Quasi-experimental pre–post design, comparing two consecutive cohorts: one receiving traditional lecture-based instruction and the next completing the redesigned PBL version. | 150 second-year PharmD students. | To describe the redesign of a large lecture-based self-care pharmacy course into a small-group, case-based, active learning course, and to evaluate its impact on student performance and skill development. | Conducted at the University of North Carolina Eshelman School of Pharmacy within the required second-year PharmD course “Patient Self-Care.” The course was restructured to shift from passive lecture delivery to small-group, student-centered learning. | - Students and faculty perceived higher motivation, accountability, and collaboration in the redesigned PBL format. - Students demonstrated enhanced teamwork, communication, and problem-solving abilities. - Minor decline noted in written communication skills due to reduced writing assignments. |
| **Ge, 2010** | United States | Mixed methods | Experimental study using a pre-post design, comparing students who received a web-based cognitive scaffolding system (question prompts, peer review, expert modelling) with a control group who did not. | 96 third-year pharmacy students (49 in the treatment group and 47 in the control group). | To investigate the effects of question prompts and peer review on scaffolding students’ PBL in a web-based cognitive support system. | Conducted at the University of Oklahoma, College of Pharmacy in collaboration with faculty teaching and the students enrolled in a clinical communications course. A cognitive support system was introduced to scaffold novice learners’ problem-solving and metacognitive skills through question prompts, peer review, and expert modelling. | - Exposure to expert modelling promoted self-reflection which helped students internalize standards of practice and improve self-directed professional development. - The support system helped students build metacognitive skills fundamental to professionalism. - The peer review mechanism encouraged students to consider multiple perspectives, fostering collaboration and openness to feedback. |
| **Giuliano, 2021** | United States | Mixed methods | Comparative study between two cohorts: traditional PBL (T-PBL, written format) and verbal defense PBL (V-PBL). Quantitative data collected via the Motivated Strategies for Learning Questionnaire (MSLQ); qualitative data from focus groups with thematic analysis. | 82 students in the traditional PBL group and 47 students in the verbal defense PBL group. Participants were third-year. | To evaluate the impact of replacing written problem-solution reports with a verbal defense format during the PBL reporting phase on pharmacy students’ motivation and learning outcomes, while maintaining all other core PBL components unchanged. | Conducted within a third-year PharmD PBL course at Wayne State University, comparing a traditional written solution format with a verbal defense format to examine how modifying the reporting phase of PBL affects student motivation, learning, and perceived authenticity of clinical reasoning. | - There were no statistically significant differences between the two formats on any MSLQ subscale, but both formats led to measurable growth in these non-clinical learning competencies. - Students increasingly believed their effort directly impacts learning outcomes — a key aspect of self-efficacy and metacognition. - Students developed better organizational skills, used more structured approaches to synthesize and explain information – aligning with cognitive organization and time management. - Significant within group improvement in critical thinking. - Instructors observed improved metacognitive awareness. |
| **Hamoudi, 2010** | United Arab Emirates | Quantitative descriptive | Descriptive cross-sectional study conducted after PBL implementation in pharmacology and therapeutics courses.  Data were collected using a structured questionnaire consisting of both Likert-scale items and open-ended questions to capture students’ perceptions and attitudes. | 95 second and third-year Bachelor of Pharmacy students. | To assess the impact of PBL on the learning behaviour of pharmacy students, particularly in terms of knowledge, skills, and attitudes, with a focus on non-clinical competencies. | Conducted over two semesters with first-year pharmacy students at RAK College of Pharmaceutical Sciences. PBL was integrated into five courses, and students came from diverse cultural and educational backgrounds. | - PBL led to significant improvements in students’ knowledge, skills, and attitudes as rated by facilitators, with baseline-to-end PBL gains statistically significant (P < 0.01). - Students highlighted that PBL increased their motivation, confidence, and engagement but noted challenges with time management, group participation balance, and facilitator variability. |
| **Hartayu, 2018** | Indonesia | Mixed methods | The study used a developmental, mixed methods design, with qualitative components including focus group discussions with faculty and preceptors, expert panel review to develop and refine training materials, and analysis of students’ reflective journals and supervisors’ observations, alongside performance assessment data. | Unspecified number of students. | To develop and evaluate training materials integrating PBL with reflective pedagogy to support pharmacy students’ competence in pharmaceutical care. | Conducted in community pharmacy internship settings in Indonesia (Yogyakarta, Solo, and Semarang), where pharmacy students undertook experiential training in pharmaceutical care using a learning model that integrated PBL with the reflective pedagogy paradigm. | - The initiating trigger cases were perceived as realistic and aligned with community pharmacy practice, and were associated with improvements in students’ problem-solving ability, critical thinking, and confidence in clinical decision-making. - Supervisors reported that the cases facilitated supervision and supported students’ development in identifying and resolving drug-related problems, formulating business and home pharmacy care plans, and communicating and collaborating effectively with other healthcare professionals. |
| **Hogan, 2006** | United States | Quantitative descriptive | Descriptive cross-sectional survey administered to two consecutive graduating cohorts (classes of 2004 and 2005). | 109 graduating PharmD students.  Class of 2004: 69 students.  Class of 2005: 40 students. | To evaluate graduating pharmacy students’ perceptions of their readiness for advanced pharmacy practice experiences (APPEs) and the role of problem-based learning in supporting this preparation. | PBL was implemented as the sole instructional approach in the third professional year of the PharmD curriculum at the University of Mississippi School of Pharmacy, with graduating students (classes of 2004 and 2005) surveyed to evaluate how this PBL-based year prepared them for their APPEs. | - 80% of students reported above-average preparedness in self-directed learning and information literacy following exposure to PBL. - Students reported mostly positive outcomes regarding improvements in their communication skills, and interprofessional communication as a consequence of PBL exposure. |
| **Hussain, 2019** | Malaysia | Quantitative descriptive | The study employed a quasi-experimental, comparative design with pre- and post-intervention assessments. Academic performance was evaluated using final exam results, and student perceptions were collected through questionnaires to assess the impact of problem-based learning. | **264 undergraduate pharmacy students** (131 in 2015, 133 in 2016). | To investigate pharmacy students’ perceptions of an industry-based PBL approach and its impact on their academic performance in drug delivery (pharmaceutics) courses. | Conducted in undergraduate pharmaceutics drug delivery courses at Universiti Teknologi MARA (Malaysia), where an industry-based PBL approach using formulation and industrial scenarios was introduced and students’ perceptions and academic performance were compared with cohorts taught by traditional tutorials. | - Student feedback was largely positive; over 80% reported that group work and presentations helped stimulate their critical thinking and problem-solving skills. - A majority of students reported positive emotions (relaxed, motivated, stimulated) after PBL sessions, reflecting increased confidence and engagement. |
| **Jones, 2005** | Australia | Qualitative | Post-intervention evaluation of PBL using student surveys and reflective self-assessment. | Not explicitly mentioned. | To evaluate whether using problem-based learning to teach a microbiology unit to pharmacy students could enhance active, self-directed learning and skill development compared with traditional lectures and practicals. | Second-year undergraduate pharmacy students at Monash University where PBL was implemented in a microbiology unit on identification of food-borne pathogens. | - Students reported increased engagement and motivation in learning in PBL compared to traditional teaching means. - Students reported that PBL supported their ability to be self-directed in their learning, being able to independently search, appraise, and synthesize literature. - Most students reported positive experiences working in groups of 3–4, with high satisfaction with collaboration and peer learning, though some expressed discomfort with unequal contribution. |
| **Juba, 2014** | United States | Mixed methods | Pre–post quasi-experimental design, with free-response pre- and post-tests, case performance scores, and thematic analysis of student self-reflections. | A total of 25 second- and third-year pharmacy students. | To design, implement, and evaluate a problem-based learning elective in pain and palliative care aimed at enhancing students’ pharmacotherapy knowledge in pain and symptom management, strengthening clinical reasoning, and fostering self-directed learning skills. | Second- and third-year Doctor of Pharmacy students enrolled in a 2-credit problem-based learning elective in pain and palliative care at St. John Fisher College, using weekly case-based discussions to teach pain and symptom management pharmacotherapy. | - Performance on three individual case studies averaged >80%, with few recurrent errors, demonstrating students’ ability to develop their clinical reasoning and application skills in complex cases. - Students demonstrated greater confidence in justifying individualized, patient-centred treatment plans in complex “grey” therapeutic situations. |
| **Lipari, 2022** | United States | Quantitative descriptive | Descriptive, pre–post observational design, evaluating changes in students’ self-assessed PBL skills and professional behaviours before and after participation in a scaffolded introductory PBL course, with comparison to facilitator assessments. | 88 first-year PharmD students. | To describe the theoretical framework underpinning the development and implementation of the introductory PBL course, and to examine students’ development of PBL skills and professional behaviour. | Conducted at the Wayne State University, College of Pharmacy and Health Sciences, introducing PBL-naïve first-year pharmacy students to a scaffolded, theory-informed introductory PBL course designed to prepare them for a longitudinal PBL sequence. | - Students’ self-assessed PBL skills (10 items) improved significantly from the first to the second assessment P < .001. |
| **Lobuteva, 2019** | Russia | Mixed methods | Cross-sectional Mixed methods design combining anonymous questionnaire surveys with focus group discussions to determine students’ perceptions of PBL and compare views across the different stages of training. | 423 students (236 first-year and 187 final-year students). | To examine the application of PBL in pharmaceutical education and to compare its perceived advantages among first- and final-year university students. | Undergraduate pharmacy at Sechenov First Moscow State Medical University to determine students’ perceptions of PBL for competency-development, with a comparative focus between first- and final-year cohorts. | - PBL enhanced the development of critical-thinking skills in students, allowing them to apply theoretical knowledge to real-life situations. - Supported the development of communication and teamwork through the group-based exercises. - Increased students’ professional readiness for life-long and self-directed learning. |
| **McKenzie, 2017** | United Kingdom | Qualitative | Qualitative exploratory design using semi-structured interviews with students and a focus group with faculty. | 7 second-year and 15 third-year MPharm students.  6 faculty members. | To explore the impact of introducing a PBL approach into a second-year pharmaceutics module, focusing on student and staff experiences, perceived learning benefits, and outcomes. | 2nd and 3rd year MPharm students at Robert Gordon University working in small groups (7–8) on mini-projects involving pharmaceutical formulation problems guided by facilitators. | - Students appreciated the independence and contextual learning benefits of PBL but reported stress and confusion during early implementation. - Faculty identified challenges with large class sizes, inconsistent supervision, and time demands. - Exam pass rates improved modestly (MDM: 79%→82%; Therapeutic Delivery: 67%→79% - PBL enhanced lifelong learning attitudes and group collaboration but required stronger structure and clearer expectations. |
| **Nasr, 2017** | Qatar | Qualitative | Descriptive faculty evaluation and reflective analysis were conducted using a SWOC (Strengths, Weaknesses, Opportunities, and Challenges) framework. Data sources included field notes, course change forms, and student feedback. Two faculty members independently reviewed the data and then reached consensus on the evaluation of the PBL implementation. | Four pharmacy faculty with PBL experience and 75 female BSc (Pharm) students across three cohorts (approximately 25 per year: second, third, and final year). | To describe and critically evaluate the implementation of PBL within a Canadian-accredited undergraduate pharmacy curriculum in a Middle Eastern context, and to identify cultural and contextual factors influencing its success. | Conducted at the College of Pharmacy, Qatar University; PBL was first introduced in 2014 within a final-year integrated case-based learning course and later expanded to earlier years of study. | - PBL enhanced learning engagement, collaboration, and alignment with accreditation standards. Students appreciated teamwork and independent learning opportunities. - Weaknesses: Inconsistent facilitation, inadequate student preparation, and limited logistical support. - Resistance to active learning among students, high faculty workload, and cultural norms (e.g., high power distance and preference for didactic instruction) limited acceptance and effective adoption of PBL. |
| **Nicholl, 2012** | Canada | Mixed methods | Interventional design using a cross-sectional evaluation, with outcomes assessed based on students’ performance and course evaluation surveys. | 239 fourth-year pharmacy students. | To implement and evaluate a model for small-group PBL within a large class environment, facilitated by a single instructor. | Fourth-year pharmacy students enrolled in a pharmaceutical sciences course at the Faculty of Pharmaceutical Sciences, University of British Columbia. The study examined how small-group PBL supported the development of clinical reasoning and teamwork skills, drawing on course evaluations from seven classes conducted over a five-year period (2006–2010). | - Majority of respondents noted that PBL strengthened clinical reasoning and problem solving, whilst promoting them to be self-directed and autonomous in their learning. - Students reported enhanced communication and teamwork skills following exposure to PBL, with better engagement in small-group discussions, peer feedback, and collaborative decision-making. |
| **Poh-Yen, 2017** | Malaysia | Quantitative descriptive | Experimental one-group design (n=5) with multiple performance assessments across a 14-week research project. The PBLab group’s results were compared descriptively to traditional supervision. | 5 second year students and 1 group facilitator | To evaluate the outcomes and benefits of the Problem-Based Laboratory (PBLab) approach focusing on improving novice researchers’ research skills, teamwork, and leadership. | Second-year Diploma in Pharmacy students conducting a chemistry-based laboratory research project under a trained facilitator. | - Students reported high satisfaction with the PBLab method and facilitator support. - Students demonstrated significant development in research, teamwork, and leadership skills, enhanced engagement, accountability, and reflection. - Students expressed increased independence, motivation and confidence for future research projects. |
| **Raisch, 1995** | United States | Quantitative descriptive | Students were randomly assigned to PBSCL (experimental, n = 26) or traditional externship (control, n = 19) groups. Both groups completed the same final examination and survey. | 45 fourth-year pharmacy students.  14 facilitators. | To develop, implement, and evaluate a problem-based, student-centered learning (PBSCL) program within pharmacy externship rotations, and to assess its impact on students’ knowledge and problem-solving abilities compared to traditional externship experiences. | Conducted at the University of New Mexico College of Pharmacy during clinical externship rotations in both hospital and community pharmacy settings. Ten preceptors were trained as PBL facilitators. All participants were completing externship rotations as part of the final stage of their pharmacy degree. | - PBSCL students scored significantly higher on knowledge and problem-solving case-based exams. - PBSCL improved students’ problem-solving skills, application of didactic knowledge, teamwork, and self-directed learning. |
| **Ravari, 2014** | Iran | Quantitative descriptive | Quasi-experimental self-control with a pre-post educational intervention design, in which the same cohort of pharmacy students experienced traditional laboratory teaching in one semester and a modified PBL in the following semester. | 73 pharmacy students. | To assess a modified PBL approach in medicinal chemistry laboratories, and to evaluate its effectiveness, benefits, and drawbacks, with a particular focus on its impact on student engagement and learning outcomes. | Undergraduate medicinal chemistry laboratory course at Kerman University Medical Sciences to evaluate the introduction of a PBL approach in a medical chemistry course to support students’ learning and skills development in laboratory work. | - Around two-thirds of students found the modified PBL to improve motivation, self-directed learning, comprehension and dynamic problem-solving skills. However, more than half also found it anxiety inducing. - Enhanced the application of knowledge and technical competence, leading to fewer laboratory errors, and improved ability to plan and perform experiments. |
| **Winslade, 1994** | Canada | Quantitative descriptive | Course redesign and evaluation study pre-post implementation  Data collected through formal student surveys (CIEQ), informal end-of-year questionnaires, faculty feedback, and national board exam performance | 160 final-year pharmacy students.  12 trained facilitators. | To revise and evaluate a large-group therapeutics course by incorporating PBL and pharmaceutical care principles to improve clinical reasoning, written communication, and self-directed learning. | Conducted in a large final-year undergraduate therapeutics course at the University of Toronto that was redesigned from traditional lectures to a pharmaceutical care-based PBL format to better prepare students for managing drug-related problems in practice. | - Faculty and students reported high satisfaction with the PBL format, noting enhanced teamwork and engagement and perceived improvements in student learning and written communication. - Positive student perceptions, with 73% rating the course as good to excellent and 80% indicating they would take another course delivered using this PBL format. |
| **Wright, 2014** | United Kingdom | Mixed methods | Cross-sectional questionnaire using an accreditation-informed survey by the Royal Pharmaceutical Society of Great Britain and Quality Assurance Agency with Likert-scale and open-ended questions. | 201 pharmacy students across four professional year levels. | To evaluate undergraduate pharmacy students’ perceptions of PBL and its effectiveness in developing the skills and competencies required to meet UK professional accreditation standards. | Conducted in an undergraduate Master of Pharmacy (MPharm) program at the University of East Anglia, where PBL was used across the first three years of the curriculum. | - Most students reported that PBL supported their oral communication (89.1%), team-working (83.1%), and problem-solving skills (61.7%). - PBL improved students’ ability to be self-directed and manage their own learning (67.6%). - Male students and those who preferred to work in team-based environments reported greater preference to PBL compared to other cohorts of students. |
